# Screening and Brief Interventions for Alcohol Use During Pregnancy: Practices Among US Primary Care Clinicians, DocStyles 2019

**DOI:** 10.5888/pcd20.220226

**Published:** 2023-04-13

**Authors:** Caitlin Green, Nisha George, Youngjoo Park, Clark H. Denny, Mary Kate Weber, Dana Meaney-Delman, Shin Y. Kim

**Affiliations:** 1 Division of Birth Defects and Infant Disorders, Centers for Disease Control and Prevention, Atlanta, Georgia; 2 Eagle Global Scientific, LLC, Atlanta, Georgia; 3 Oak Ridge Institute for Science and Education, Oak Ridge, Tennessee

## Abstract

**Introduction:**

Alcohol use during pregnancy can cause birth defects and developmental disabilities. From 2018 through 2020, 13.5% of pregnant women reported current drinking. The US Preventive Services Task Force recommends evidence-based tools (eg, AUDIT-C and SASQ) for implementing screening and brief interventions to reduce excessive alcohol use among adults, including pregnant people, for whom any alcohol use is considered excessive.

**Methods:**

We used DocStyles 2019 data to conduct a cross-sectional analysis to examine current screening and brief intervention practices that primary care clinicians conduct among pregnant patients; clinicians’ confidence levels in conducting screening, brief interventions, and referral to treatment; and the documentation of brief interventions in the medical record.

**Results:**

A total of 1,500 US adult medicine clinicians completed the entire survey. Among the respondents who conduct screening (N = 1,373) and brief interventions (N = 1,357) in their practice, nearly all reported implementing screening (94.6%) and brief interventions (94.9%) with their pregnant patients for alcohol use, but fewer than half felt confident about conducting their screening practices (46.5%). Two-thirds (64%) reported using a tool that met the criteria recommended by the US Preventive Services Task Force (USPSTF). Over half documented brief interventions in electronic health record notes (51.7%) or designated space (50.7%).

**Conclusion:**

Pregnancy presents a unique opportunity for clinicians to incorporate screening into routine obstetric care and encourage behavior change among patients. Most providers reported always screening their pregnant patients for alcohol use, but fewer used evidence-based USPSTF-recommended screening tools. Increased clinician confidence in screening and brief intervention, the use of standardized screening tools tailored to pregnant people, and maximal use of electronic health record technology may enhance the benefits of their application to alcohol use, which ultimately can reduce adverse outcomes associated with alcohol use during pregnancy.

**Summary**

**What is already known on this topic?**

Prenatal alcohol exposure can result in fetal alcohol spectrum disorders. Screening for alcohol use is recommended for women who receive obstetric–gynecologic care annually and during the first trimester of pregnancy.

**What is added by this report?**

Although the primary care clinicians in this study reported screening their pregnant patients for alcohol use, fewer than half reported feeling confident in their screening and brief intervention.

**What are the implications for public health practice?**

Resources to support the implementation of alcohol screening and brief interventions, including electronic health record–based clinical decision-making tools, may improve clinicians’ confidence and use of these tools.

## Introduction

Excessive alcohol use is associated with more than 60 disease conditions among all adults (1). Any alcohol use during pregnancy is considered excessive (2) and can cause birth defects and developmental disabilities known as fetal alcohol spectrum disorders (3). From 2018 through 2020, 13.5% of pregnant women reported current drinking, and 5.2% reported binge drinking in the past 30 days (4). There is no known safe amount of alcohol use during pregnancy (5,6). (Hereinafter, for clarity in terminology, “pregnant women” is used for consistency with a cited article, but otherwise we use “pregnant people” or “pregnant patients” to be inclusive of those who are pregnant but do not identify as women.)

The American College of Obstetricians and Gynecologists recommends that all women who receive obstetric–gynecologic care be screened for alcohol use annually and during the first trimester of pregnancy (7). The US Preventive Services Task Force (USPSTF) recommends screening and brief interventions to reduce excessive alcohol use among adults, including pregnant people for whom any alcohol use is considered excessive (8). In their updated 2018 recommendations, the USPSTF determined that screening tools of 1 to 3 items have the best accuracy for assessing adults, such as the abbreviated Alcohol Use Disorders Identification Test-Consumption (AUDIT-C) and the National Institute on Alcohol Abuse and Alcoholism-recommended Single Alcohol Screening Question (SASQ) (8). Although some studies have examined current screening and intervention practices among primary care clinicians, few have focused on pregnant people. Moreover, little is known about how brief intervention practices are documented in the medical record. This study examined current screening and brief intervention practices conducted by primary care clinicians for pregnant patients; clinicians’ confidence levels in conducting screenings, brief interventions, and referrals to treatment; and the documentation of brief interventions in the medical record.

## Methods

DocStyles is a web-based survey developed by Porter Novelli Public Services (9) and conducted by Sermo, an online global network comprising more than 350,000 medical professionals (10). The survey is administered twice a year (spring and fall) to clinicians registered with Sermo, including family physicians, internists, obstetricians/gynecologists (OB/GYNs), pediatricians, nurse practitioners, and physician assistants. Survey respondents included only those who practiced in individual, group, or hospital settings in the US and who had been practicing for at least 3 years. Depending on clinical specialty, the survey length is 6 to 33 minutes. Of 2,696 clinicians who received the 2019 survey, 1,750 (65%) completed it by the deadline; respondents who completed the survey were paid an honorarium. This study was deemed nonresearch and did not require institutional review board or Office of Management and Budget review.

To examine clinicians’ practices in screening and conducting brief interventions for alcohol use during pregnancy, respondents were asked 5 questions (Appendix). Of the 1,750 clinicians who completed the survey, pediatricians (n = 250) were exempt from answering our questions because they generally do not treat pregnant people. Thus, a total of 1,500 clinicians were included in our analysis. Two questions were designed to obtain information on how frequently clinicians screened and provided brief interventions for alcohol use among pregnant patients, with answer options ranging on a Likert scale from “always” to “never.” Respondents were also asked to indicate their level of confidence in conducting screening and brief intervention, ranging on a Likert scale from “very confident” to “not at all confident.” Clinicians were asked to indicate which of the following screening tools they used in their practice: Parents, Partner, Past, and Present (4P’s) (11), Parents, Partner, Past, Pregnancy (4P’s Plus) (12), and Parents, Peers, Partner, Pregnancy, Past (5P’s) (13); Alcohol, Smoking, and Substance Involvement Screening Test (ASSIST) (14) or Alcohol, Smoking, and Substance Involvement Screening - Frequency and Concerns (ASSIST-FC) (15); AUDIT (16) or AUDIT-C (17); Cut down, Annoyed, Guilty, Eye-opener (CAGE) (18); National Institute on Drug Abuse (NIDA) Quick Screen (19); SASQ (20); and Tolerance, Annoyed, Cut down, Eye-opener (T-ACE) (21) or Tolerance, Worried, Eye-opener, Amnesia, Kut down (TWEAK) (22). Finally, clinicians were asked to indicate how they document brief interventions in patient records (eg, electronic health record [EHR] or paper record). Frequency of responses to these questions was calculated and stratified by clinician type. The total number of screening tools used (eg, ≥1, ≥2) was assessed. We used SAS 9.4 (SAS Institute Inc) to conduct a cross-sectional analysis.

## Results

A total of 1,500 clinicians were queried in 2019: 414 family physicians, 586 internists, 250 OB/GYNs, 142 nurse practitioners, and 108 physician assistants. Of the 1,500 surveyed clinicians, 127 indicated “not applicable” for screening pregnant people and 143 indicated “not applicable” for conducting brief interventions; these respondents were excluded from this analysis. Most clinicians (94.6%) reported screening pregnant patients for alcohol use, with over half reporting screening always (67.2%), 15.9% screening often, 11.5% screening sometimes, and 5.4% never screening (Table 1). Additionally, more than half reported always conducting brief interventions with their pregnant patients (66.0%), 18.8% reported often, 10.1% reported sometimes, and 5.1% reported never conducting brief interventions (Table 2). Data by clinical specialty indicated that clinicians with the highest proportions of always screening and always conducting brief interventions with their pregnant patients were nurse practitioners (72.7% and 74.3%, respectively) and OB/GYNs (80.8% and 68.6%, respectively). Physician assistants had the highest proportion of never screening (10.2%) or conducting brief interventions (8.4%). 

**Table 1:** Frequency, Clinician Screening of Pregnant Patients for Alcohol Use, DocStyles, 2019^a^

Clinician specialty	Never	Sometimes	Often	Always
Family physician (n = 376)	20 (5.3)	42 (11.2)	60 (16.0)	254 (67.6)
Internist (n = 542)	33 (6.1)	71 (13.1)	101 (18.6)	337 (62.2)
Obstetrician/gynecologist (n = 240)	5 (2.1)	20 (8.3)	21 (8.8)	194 (80.8)
Nurse practitioner (n = 117)	6 (5.1)	8 (6.8)	18 (15.4)	85 (72.7)
Physician assistant (n = 98)	10 (10.2)	17 (17.4)	18 (18.4)	53 (54.1)
Total (N = 1,373)	74 (5.4)	158 (11.5)	218 (15.9)	923 (67.2)

a Porter Novelli. DocStyles. http://styles.porternovelli.com/docstyles (9). Respondents who selected “not applicable” to screening pregnant patients were excluded (n = 127). Values are number (percentage).

**Table 2:** Frequency, Clinician Brief Interventions With Pregnant Patients Who Screen Positive for Risky Alcohol Use, DocStyles, 2019^a^

Clinician specialty	Never	Sometimes	Often	Always
Family physician (n = 377)	20 (5.3)	30 (8.0)	76 (20.2)	251 (66.6)
Internist (n = 533)	25 (4.7)	63 (11.9)	100 (18.8)	345 (64.7)
Obstetrician/gynecologist (n = 239)	9 (3.8)	22 (9.2)	44 (18.4)	164 (68.6)
Nurse practitioner (n = 113)	7 (6.2)	10 (8.9)	12 (10.6)	84 (74.3)
Physician assistant (n = 95)	8 (8.4)	12 (12.6)	23 (24.2)	52 (54.7)
Total (N = 1,357)	69 (5.1)	137 (10.1)	255 (18.8)	896 (66.0)

a Porter Novelli. DocStyles. http://styles.porternovelli.com/docstyles (9). Respondents who selected “not applicable” to conducting brief interventions with pregnant patients were excluded (n = 143). Values are number (percentage).

Most clinicians (87.7%) who reported screening their pregnant patients for alcohol use reported using 1 to 4 screening tools, with 44% using 1 or 2 tools and 43.7% using 3 or 4 tools. Another 3.9% reported screening their pregnant patients but did not use any screening tools. The 2 most reported screening methods were a single question about the number of days per week at least 1 alcoholic drink was consumed (60.7%) and the CAGE tool (58.6%) (Table 3). With respect to the brief screening tools that meet the criteria recommended by USPSTF, 44.6% of providers reported using the SASQ tool and 19.4% used the AUDIT or AUDIT-C tool. Between these 2 tools, 34% of providers reported using SASQ but not AUDIT or AUDIT-C, and 10% reported using AUDIT or AUDIT-C but not SASQ; 8.4% reported using both tools. Few providers reported using T-ACE or TWEAK (6.3%); 4P’s, 4P’s Plus, or 5P’s (4.5%); ASSIST or ASSIST-FC (2.5%); or the NIDA Quick Screen (3.0%).

**Table 3:** Screening Methods of Primary Care Clinicians for Alcohol Use During Pregnancy, by Clinician Specialty – DocStyles, 2019^a^

Screening method	Family physician (n = 414)	Internist (n = 586)	Obstetrician/gynecologist (n = 250)	Nurse practitioner (n = 142)	Physician assistant (n = 108)	Total (N = 1,299^b^)
Screening tools						

4 Ps, 4Ps Plus, or 5 Ps	12 (0.9)	21 (1.6)	15 (1.2)	6 (0.5)	5 (0.4)	59 (4.5)
ASSIST or ASSIST-FC	12 (0.9)	15 (1.2)	2 (0.2)	3 (0.2)	1 (0.1)	33 (2.5)
AUDIT or AUDIT-C	92 (7.1)	108 (8.3)	16 (1.2)	20 (1.5)	16 (1.2)	252 (19.4)
CAGE	236 (18.1)	326 (25.1)	88 (6.8)	59 (4.5)	52 (4.0)	761 (58.6)
NIDA Quick Screen	17 (1.3)	15 (1.2)	2 (0.2)	1 (0.1)	4 (0.3)	39 (3.0)
SASQ	158 (12.2)	226 (17.4)	100 (7.7)	56 (4.3)	39 (3.0)	579 (44.6)
T-ACE or TWEAK	15 (1.2)	26 (2.0)	37 (2.9)	3 (0.2)	1 (0.9)	82 (6.3)

Alcohol-related questions						

Ask number of drinking days per week	206 (15.9)	298 (22.9)	157 (12.1)	71 (5.5)	56 (4.3)	788 (60.7)
Ask number of drinks per occasion	196 (15.1)	272 (20.9)	134 (10.3)	69 (5.3)	56 (4.3)	727 (56.0)
Some other screening method	4 (0.3)	7 (0.5)	4 (0.3)	5 (0.4)	0	20 (1.5)

Abbreviations: 4Ps: Parents, Partner, Past, Present; 4Ps Plus: Parents, Partner, Past, Pregnancy; 5Ps: Parents, Peers, Partner, Pregnancy, Past; ASSIST: Alcohol, Smoking and Substance Involvement Screening Test; ASSIST-FC: Alcohol, Smoking and Substance Involvement Screening Test–Frequency & Concern; CAGE: Cut down, Annoyed, Guilty, Eye-opener; SASQ: Single Alcohol Screening Question; T-ACE: Tolerance, Annoyed, Cut down, Eye-opener; TWEAK: Tolerance, Worried, Eye-opener, Amnesia, Kut down.

a Porter Novelli. DocStyles. http://styles.porternovelli.com/docstyles (9).

b Respondents who selected “not applicable” or “never screen” pregnant patients were excluded (n = 201). Respondents were able to select more than one screening tool. Values are number (percentage).

Fewer than half of all clinicians surveyed reported feeling very confident in screening for excessive alcohol use (46.5%). Additionally, about one-third (34.9%) reported feeling very confident in conducting brief interventions for alcohol problems with their pregnant patients. Of clinician specialties, OB/GYNs reported the highest proportion of feeling very confident in both screening (56.1%) and conducting brief interventions (40.3%) for alcohol use among pregnant patients. More than half of clinicians surveyed documented brief intervention practices by using EHR notes (51.7%) or a designated space in the EHR (50.7%), with 14.4% of providers using both the EHR notes and designated space. Of the provider specialties, nurse practitioners and family physicians reported the highest documentation in the EHR designated space (55.6% and 51.7%, respectively), and internists and family physicians reported the highest documentation in EHR notes or other space (54.3% and 53.4%, respectively). Among clinicians who always conducted brief interventions with pregnant patients, more than half documented it in the EHR (55.5% in a designated space and 51.9% in notes), and few documented it in the paper record (6.1% in the designated space and 8.2% in notes).

## Discussion

Our analysis describes self-reported screening and brief intervention practices to assess alcohol use during pregnancy reported by a sample of US primary care clinicians. Although almost all clinicians reported screening their pregnant patients for alcohol use and two-thirds reported routinely conducting brief interventions, fewer than half felt confident in their screening and brief intervention, and two-thirds used an evidence-based tool that met USPSTF-recommended criteria. Few providers reported using tools specifically tailored to pregnancy (ie, T-ACE, TWEAK, 4Ps, 4Ps Plus, 5Ps). Clinicians may use specific screening tools, depending on the patient population for which they provide care. Notably, a few who reported screening their pregnant patients indicated not using any screening tool, and only half who screened used a USPSTF-recommended screening tool. Almost all reported recording brief interventions in EHRs in either a designated space or in notes. These findings are consistent with other studies that reported population-based self-reported data on alcohol screening and brief interventions among primary care clinicians (23,24). 

The USPSTF recommends using evidence-based brief screening tools to assess adults, including pregnant patients, for excessive alcohol use (8). Pregnancy presents a unique time for behavior change because pregnant people with substance use disorders may feel more motivated to seek treatment to potentially benefit themselves and their child (25,26). Primary care clinicians can use this opportunity to incorporate screening services into routine obstetric care and provide specialized care and treatment if needed (27). Evidence shows that brief screening tools (eg, AUDIT-C and SASQ) with high sensitivity, low specificity, and 1 to 3 items are useful as an initial indication of excessive drinking behavior, and USPSTF recommends further follow-up with a more in-depth tool with greater specificity (eg, AUDIT) (8). Using AUDIT-C as the initial screener could facilitate an easier transition to the full AUDIT and may be considered by clinicians as their preferred tool because it allows for an in-depth risk assessment to inform care for those at risk. Screening tools specifically tailored to pregnancy may be considered a preferred tool for pregnant patients after further review and validation of test accuracy (8,28,29). Stigma surrounding alcohol use during pregnancy and the fear of prosecution or having children removed from their care may deter patients from seeking help and, in turn, may contribute to their underreporting alcohol use when screened by their clinicians (30). The use of nonstigmatizing language coupled with evidence-based screening tools can facilitate a safer environment for pregnant patients to answer questions and receive the appropriate care they need (31). 

Additionally, using screening tools that can assess a broad spectrum of alcohol use problems (ie, mild to severe) can lead to more opportunities for clinicians to conduct appropriate brief interventions or provide referrals to treatment of the patient. A 2020 cross-sectional study surveyed family physicians, midwives, and obstetricians practicing in Canada and found that although nearly all clinicians reported screening pregnant women for alcohol use, brief interventions were not conducted as widely (32), which is consistent with the results of our analysis. USPSTF recommends that a brief intervention lasts 6 to 15 minutes and facilitates tailored feedback about the patient’s risks and potential consequences of their current drinking habits (23,33). Multiple brief intervention sessions reduce patients’ alcohol consumption more effectively, increase adherence to drinking guidelines, and increase the likelihood of abstaining completely from drinking while pregnant (8,23). Electronic (ie, cell phones or tablets) or computer-based approaches to brief interventions can be alternatives to person-delivered interventions and may require less clinician training and time commitment (34). Some studies implementing brief electronic interventions have shown promising results in reducing alcohol use among pregnant patients (35,36). Our study examined the frequency of brief interventions conducted by primary care clinicians, but more information is needed to understand which brief intervention approaches are most often used and should be considered as the focus in future studies. 

The frequency and type of documentation for brief interventions in the patient medical record is not known. Almost all clinicians who responded to the DocStyles survey reported use of patient EHRs to document brief interventions with half reporting use of a designated space and the other half reporting use of notes or other space. Using electronic health records instead of paper records has various advantages, including increasing each clinician’s ability to better manage care for their patients, providing more accurate and complete information about patients at the point of care, and enabling more accurate and effective diagnoses (37). The adoption of EHRs in office-based clinics more than doubled from 2008 (42%) to 2017 (85.9%) (38). Yet there is still variability in the design of EHR systems, which can lead to inconsistencies in how and what information is captured. More information may assist in understanding current documentation practices within EHRs and facilitate standardized and consistent documentation practices. 

Standards-based clinical decision support tools have been developed to assist providers in integrating screening tools (eg, AUDIT) and brief intervention aids into their EHR system (39). The frequency of EHR integration and use of these tools is not yet known. Additionally, a recent cross-sectional study identified resources that US primary care clinicians believe would be helpful in their screening and brief intervention practice, and noted resources on implementation, patient education materials, and referral information (24). 

### Limitations

The findings of our study are subject to at least 4 limitations. First, DocStyles data are self-reported, making the results subject to social desirability and recall bias, which may have led to overreporting of the implementation of screening and brief intervention practices. Second, only clinicians registered with Sermo were eligible to be in the sample, thus making results subject to selection bias. Third, our sample of respondents was not representative of all clinicians in their respective specialty, and data were not weighted, so the percentages reported for the total sample are not nationally representative. Lastly, the question regarding screening tools was not a comprehensive list of all possible tools for screening for excessive alcohol use. 

### Conclusion

Pregnancy presents a unique opportunity for primary care clinicians to incorporate screening into routine obstetric care and encourage behavior change among patients (25–27). Most clinicians reported always screening their pregnant patients for alcohol use, but fewer used evidence-based USPSTF-recommended screening tools and tools tailored to pregnant patients. Resources to support the implementation of alcohol screening and brief interventions, including EHR-based clinical decision support tools, may improve clinician confidence in and use of these tools. Increased confidence in the use of standardized screening tools tailored to pregnant people and maximal use of EHR technology may enhance the benefits of screening and brief intervention for alcohol use, which could reduce adverse outcomes associated with alcohol use during pregnancy.

## Appendix DocStyles^a^ Questions and Answer Options

**Table:**

**1. How often are pregnant people screened for alcohol use in your practice?** *Never; Sometimes; Often; Always; Not applicable*
**2. Among pregnant people who screen positive for risky alcohol use, how often are brief interventions conducted?** *Never; Sometimes; Often; Always; Not applicable*
**3. How confident do you feel about your ability to do each of the following?** *(Select one response per item: Not at all confident; A little confident; Fairly confident; Very confident; Not applicable)* • Identify risky alcohol use for pregnant patients • Conduct brief interventions for alcohol problems for pregnant patients • Refer pregnant patients to treatment for alcohol problems • Identify risky alcohol use for adult patients • Conduct brief interventions for alcohol problems for adult patients • Refer adult patients to treatment for alcohol problems
**4. How do you screen for alcohol misuse in pregnancy? ** *(Select all that apply)* • AUDIT or AUDIT-C • CAGE • T-ACE or TWEAK • 4 Ps, 4Ps Plus, or 5 Ps • NIDA Quick Screen • ASSIST or ASSIST-FC • Ask binge question/single question screener (SASQ) (eg, “How many times in the past year have you had 4 (women) / 5 (men) or more drinks in a day?”) • Ask number of drinks per occasion (eg, “On a typical drinking day, how many drinks do you have?”) • Ask frequency of drinking (eg, “On average, how many days a week do you have an alcoholic drink?”) • Some other screening method • I don’t screen
**5. In your practice, how is a brief intervention for alcohol misuse documented?** *(Select all that apply)* • Electronic health record (EHR)–designated space • EHR – in notes or other space • EHR – designated space and notes or other space^b^ • Paper record – in a designated space • Paper record – in notes or other space • Paper record – designated space and noted or other space^a^ • No documentation • Other

Abbreviations: 4Ps, Parents, Partner, Past, and Present (11); 4Ps, Plus, Parents, Partner, Past, Pregnancy (12); 5Ps, Parents, Peers, Partner, Pregnancy, Past (13); ASSIST. Alcohol, Smoking, and Substance Involvement Screening Test (14); ASSIST-FC, Alcohol, Smoking, and Substance Involvement Screening Test - Frequency and Concerns (15); AUDIT, Alcohol Use Disorders Identification Test (16); AUDIT-C, Alcohol Use Disorders Identification Test - Consumption (17); CAGE, Cut down, Annoyed, Guilty, Eye-opener (18); NIDA, National Institute on Drug Abuse (19); SASQ, Single Alcohol Screening Question (20); T-ACE, Tolerance, Annoyed, Cut down, Eye-opener (21); TWEAK, Tolerance, Worried, Eye-opener, Amnesia, Kut down (22).

^a ^Porter Novelli. DocStyles. http://styles.porternovelli.com/docstyles (9).

^b^ Created variables.
